# NK cell frequencies, function and correlates to vaccine outcome in BNT162b2 mRNA anti-SARS-CoV-2 vaccinated healthy and immunocompromised individuals

**DOI:** 10.1186/s10020-022-00443-2

**Published:** 2022-02-08

**Authors:** Angelica Cuapio, Caroline Boulouis, Iva Filipovic, David Wullimann, Tobias Kammann, Tiphaine Parrot, Puran Chen, Mira Akber, Yu Gao, Quirin Hammer, Benedikt Strunz, André Pérez Potti, Olga Rivera Ballesteros, Joshua Lange, Jagadeeswara Rao Muvva, Peter Bergman, Ola Blennow, Lotta Hansson, Stephan Mielke, Piotr Nowak, Gunnar Söderdahl, Anders Österborg, C. I. Edvard Smith, Gordana Bogdanovic, Sandra Muschiol, Fredrika Hellgren, Karin Loré, Michal J. Sobkowiak, Giorgio Gabarrini, Katie Healy, Margaret Sällberg Chen, Evren Alici, Niklas K. Björkström, Marcus Buggert, Per Ljungman, Johan K. Sandberg, Soo Aleman, Hans-Gustaf Ljunggren

**Affiliations:** 1 Department of Medicine Huddinge, Center for Infectious Medicine, Karolinska Institutet, Karolinska University Hospital, 141 52 Stockholm, Sweden; 2 Department of Infectious Diseases, Karolinska University Hospital, Stockholm, Sweden; 3 Department of Laboratory Medicine, Clinical Microbiology, Karolinska Institutet, Stockholm, Sweden; 4 Department of Clinical Science, Intervention and Technology, Karolinska Institutet, Stockholm, Sweden; 5 Department of Transplantation, Karolinska University Hospital, Stockholm, Sweden; 6 Department of Oncology-Pathology, Karolinska Institutet, Stockholm, Sweden; 7 Department of Hematology, Karolinska University Hospital, Stockholm, Sweden; 8 Department of Laboratory Medicine, Biomolecular and Cellular Medicine, Karolinska Institutet, Stockholm, Sweden; 9 Department of Cellular Therapy and Allogeneic Stem Cell Transplantation (CAST), Karolinska University Hospital Huddinge, Stockholm, Sweden; 10 Department of Medicine Huddinge, Infectious Diseases, Karolinska Institutet, Stockholm, Sweden; 11 Laboratory for Molecular Infection Medicine Sweden MIMS, Umeå University, Umeå, Sweden; 12 Department of Clinical Microbiology, Karolinska University Hospital, Stockholm, Sweden; 13 Department of Microbiology, Tumor and Cell Biology, Karolinska Institutet, Stockholm, Sweden; 14 Department of Medicine Solna, Karolinska Institutet, Stockholm, Sweden; 15 Department of Dental Medicine, Karolinska Institutet, Stockholm, Sweden; 16 Department of Medicine Huddinge, Hematology and Regenerative Medicine, Karolinska Institutet, Stockholm, Sweden; 17 Department of Laboratory Medicine, Translational Research Center Karolinska (TRACK), Karolinska Institutet, Stockholm, Sweden

**Keywords:** Anti-SARS-CoV-2 antibodies, BNT162b2 mRNA vaccine, Clinical trial, COVID-19, NK cells, Innate immunity, mRNA vaccine, NKG2C, SARS-CoV-2

## Abstract

Adaptive immune responses have been studied extensively in the course of mRNA vaccination against COVID-19. Considerably fewer studies have assessed the effects on innate immune cells. Here, we characterized NK cells in healthy individuals and immunocompromised patients in the course of an anti-SARS-CoV-2 BNT162b2 mRNA prospective, open-label clinical vaccine trial. See trial registration description in notes. Results revealed preserved NK cell numbers, frequencies, subsets, phenotypes, and function as assessed through consecutive peripheral blood samplings at 0, 10, 21, and 35 days following vaccination. A positive correlation was observed between the frequency of NKG2C^+^ NK cells at baseline (Day 0) and anti-SARS-CoV-2 Ab titers following BNT162b2 mRNA vaccination at Day 35. The present results provide basic insights in regards to NK cells in the context of mRNA vaccination, and have relevance for future mRNA-based vaccinations against COVID-19, other viral infections, and cancer.

*Trial registration*: The current study is based on clinical material from the COVAXID open-label, non-randomized prospective clinical trial registered at EudraCT and clinicaltrials.gov (no. 2021–000175-37). Description: https://clinicaltrials.gov/ct2/show/NCT04780659?term=2021-000175-37&draw=2&rank=1.

**Supplementary Information:**

The online version contains supplementary material available at 10.1186/s10020-022-00443-2.

## Background

COVID-19 vaccination programs have targeted more than 60% of the global population (https://ourworldindata.org/covid-vaccinations). This achievement has been the result of major efforts by individual scientists and industries, successful outcomes of clinical trials, and subsequent global vaccination programs. Clinical trials with anti-SARS-CoV-2 vaccines have focused largely on safety and efficacy in terms of primarily antibody (Ab) responses as well as associated protection against infection and severe disease (Baden et al. 2021; Polack et al. 2020). Exploratory studies from clinical trials and other clinical studies have added significant additional insights into T cell immune responses to vaccination (Sette and Crotty 2021). In contrast, significantly less knowledge has been gained with respect to the effects on other parts of the immune system, including innate immune cells.

Belonging to the family of innate lymphoid cells, natural killer (NK) cells play an important role in controlling several human viral infections (Bjorkstrom et al. 2021). In context of vaccination, they cooperate with vaccine-elicited Abs to prevent infection (Rydyznski and Waggoner 2015). Furthermore, the ability of NK cells to regulate other cells of the immune system has been increasingly recognized (Waggoner et al. 2011). In the present context, they have been demonstrated to contribute to the regulation of vaccine-elicited T cell and B cell responses (Wagstaffe et al. 2018; Cox et al. 2021). Additionally, NK cells isolated from healthy individuals are now used as therapeutic agents in clinical trials involving human malignant (Ljunggren and Malmberg 2007; Myers and Miller 2021) and viral diseases including COVID-19 (Market et al. 2020; Soleimanian and Yaghobi 2020).

Few studies have characterized NK cells in the context of current global COVID-19 vaccination programs. Because of this, we here addressed the effects of BNT162b2 mRNA vaccination on NK cells. We took advantage of clinical material from a prospective open-label clinical trial, COVAXID, in which the safety and efficacy of the BNT162b2 mRNA vaccine was tested in healthy individuals and selected groups of immunocompromised patients (Bergman et al. 2021). In the course of the clinical trial, we directed exploratory studies towards possible effects of vaccination on NK cells, including cell numbers, frequencies, subsets, phenotypes and function. The clinical trial also allowed us to address whether the studied NK cell parameters or other traits at baseline correlated with anti-SARS-CoV-2 Ab titers following BNT162b2 mRNA vaccination. The results provide select insights into NK cells in the context of BNT162b2 mRNA vaccination, and may also be of importance for future mRNA-based vaccines against SARS-CoV-2 and other viral infections as well as against human cancer.

## Materials and methods

A list of all reagents and materials used for the present study including the corresponding RRID numbers is shown in Additional file 1: Table S1.

### Clinical trial study design

**Fig. 1:**
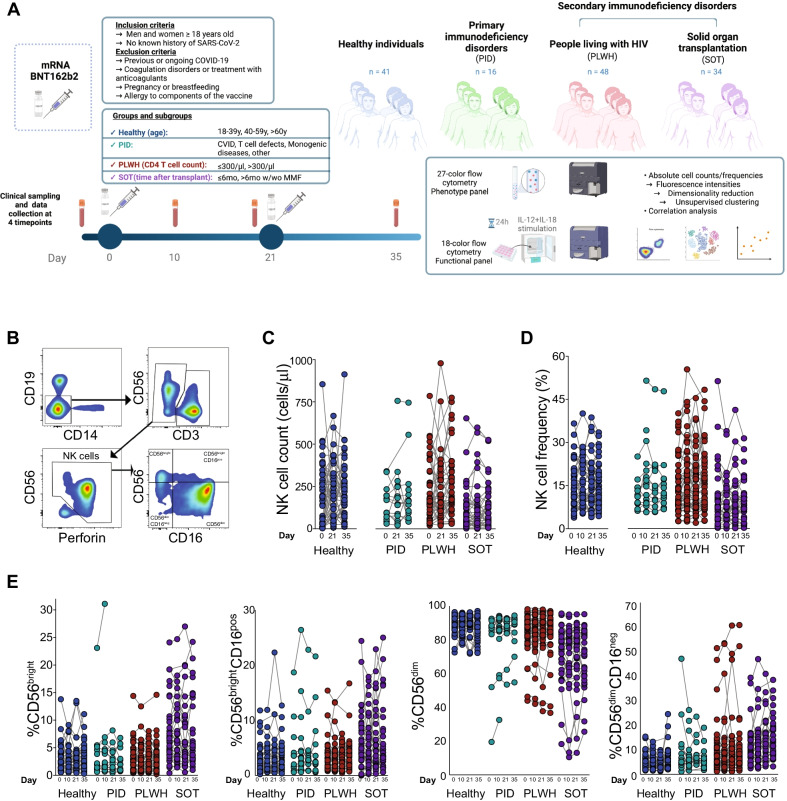
NK cell frequencies and immunophenotypes after BNT162b2 mRNA vaccine administration. **A** Study design, experimental and analytical workflow on PBMC samples from healthy individuals and select patient groups with immunodeficiency disorders receiving two doses of the BNT162b2 mRNA vaccine according to label. **B** Gating strategy to identify natural killer (NK) cells and their subsets by flow cytometry. **C** NK cell absolute numbers based on the basal absolute lymphocyte count at Days 0, 21 and 35 in healthy study subjects (left panel) and immunocompromised patients (right panels). **D** Frequency of total NK cells based on the lymphocyte gate obtained by flow cytometry analysis at Days 0, 10, 21 and 35 in the healthy study subjects (left panel) and immunocompromised patients (right panels). **E** Frequency of NK cell subsets in healthy study subjects and immunocompromised patients. Healthy individuals (Day 0 n = 37, Day 10 n = 38, Day 21 n = 36, Day 35 n = 37), PLWH (Day 0 n = 48, Day 10 n = 46, Day 21 n = 44, Day 35 n = 44), PID (Day 0 n = 12, Day 10 n = 16, Day 21 n = 14, Day 35 n = 12), SOT (Day 0 n = 34, Day 10 n = 33, Day 21 n = 33, Day 35 n = 30). Statistical analysis for **C**, **D** and **E** was performed using a Kruskal–Wallis test followed by a Dunn´s multiple comparisons test. No statistical significance difference was observed within each respective group (i.e., when comparing days 0, 10, 21 and 35)

The present study was based on clinical material from the COVAXID open-label, non-randomized prospective clinical trial, in which the safety and efficacy of two doses of the BNT162b2 mRNA (Comirnaty®, Pfizer/BioNTech) vaccine were assessed in healthy individuals and selected groups of immunocompromised patients (Bergman et al. 2021). Briefly, the trial included individuals ≥ 18 years of age, with no known history of SARS-CoV-2 infection. Healthy individuals were defined as study subjects without an immunocompromising disorder or treatment, and without significant comorbidity. They were selected to represent three age groups of similar size (18–39 years, 40–59 years, and > 60 years, respectively) including both men and women in an roughly equal balance (at least 40% of each sex). Specific immunocompromised patient groups, subgroups, and numbers of study subjects included in the present analyses are depicted in Fig. 1A and Table 1. The participants were given injections of BNT162b2 mRNA vaccine in standard dose (30 µg) into the deltoid muscle of the non-dominant arm on Days 0 and 21 of the study, i.e., in a two-dose regimen according to the label. All vaccine doses were derived from the same batch. Peripheral blood samples were taken at Day 0 (before the first vaccination), and then at Days 10, 21 (before the second vaccination), and Day 35 (analysis of the primary endpoint) (Fig. 1A).

**Table 1:** Baseline characteristics of participants included in the study

	Healthy Individudals (n = 41)	Primary Immuno-deficiencies (n = 16)	People Living With HIV (n = 48)	Solid Organ Transplantation (n = 34*)
Sex, n (%)				
Men	19 (46%)	8 (50%)	26 (54%)	18 (53%)
Women	22 (54%)	8 (50%)	22 46%)	16 (47%)
Age < 65 years, n (%)	28 (68%)	13 (81%)	37 (77%)	24 (71%)
Ongoing immune-suppression, n (%)				
Corticosteroids	0	0	0	32 (94%)
Other immune-suppressive agents	0	1 (6%)	0	34 (100%)
Subgroups (n)	1. 18–39 years (n = 16) 2. 40–59 years (n = 12) 3. > 60 years (n = 13)	1. CVID (n = 3) 2. Low number or defect T-cell function (n = 8) 3. Monogenic diseases (n = 2) 4. Other with expected normal response (n = 3)	1. Latest CD4 T cell count ≤ 300 cells/μl (n = 16) 2. Latest CD4 T cell count > 300 cells/μl (n = 32)	Time after transplantation: 1. ≤ 6 mo (n = 19) with/without MMF 2. > 6 mo with MMF (n = 6) 3. > 6 mo without MMF (n = 9)

*n* number, *HIV* human immunodeficiency virus, *CVID* common variable immunodeficiency, *IgA* immunoglobulin A, *CD* cluster of differentiation, *MMF* mycophenolate mofetil.

*The different transplants in the SOT group (n = 34) were liver (n = 19), kidney (n = 14) and kidney and pancreas (n = 1)

### Processing of peripheral blood

Heparinized blood was obtained by venipuncture and peripheral blood mononuclear cells (PBMCs) were isolated using density gradient centrifugation (Lymphoprep). To isolate PBMCs, blood diluted 1:1 in PBS was layered over in Lymphoprep in a SepMate tube and centrifuged for 10 min at 1200 g. The PBMC layer was collected by pouring the content into a new 50 ml tube followed by a two-time wash with PBS. The pelleted cells were counted, and the cell viability was calculated using trypan blue staining. Cells were cryopreserved at − 80 °C in the presence of dimethyl sulfoxide (DMSO) 10 and 90% fetal bovine serum until further use.

### Flow cytometry

The staining was performed in two separate panels for (i) PBMC cell composition and phenotype, and (ii) Intracellular staining for functional characterization. Antibodies used are listed in Additional file 1: Table S2 and S3, respectively. In brief, cryopreserved PBMC were thawed and counted immediately before use. Cells were washed twice and plated at 1–2 × 10^6^ cells in a 96-well U-bottom plate in 100 µL FACS buffer. Cell surface staining (Additional file 1:Table S2) was done for 20 min at 4 °C in phosphate-buffered saline (PBS), 2 mM EDTA, and 2% fetal bovine serum. After washing, cells were fixed in Fix/Perm solution for 30 min at 4 °C and permeabilized with Perm buffer for 30 min at 4 °C (both reagents from eBioscience FoxP3/transcription factor staining buffer set-Invitrogen), followed by intracellular staining for 30 min at 4 °C. After washing, cells were ready for acquisition.

Samples were acquired on a BD FACSymphony A5 flow cytometer (BD Biosciences) with 355-, 405, 488-, 561- and 640 nm lasers, or on a BD LSRFortessa equipped with four lasers, and data were analyzed with FlowJo software v10.7.2. Single-stained compensation beads (BD Biosciences) were used to calculate compensation matrix.

### NK cell activation and intracellular staining

Thawed cryopreserved PBMC were washed and plated at 1 × 10^6^ cells in a 96 well plate in 100 µL in complete RPMI medium 1640 (Gibco) supplemented with 10% fetal bovine serum (Thermo Scientific), 1 mM L-glutamine (Invitrogen), 100 U/mL penicillin, and 50 μg/mL streptomycin (R10 medium). Anti-human CD107a was added to the culture and cells were stimulated with IL-12 (10 ng/mL) and IL-18 (100 ng/mL) for 24 h at 37 °C and 5% CO_2_. Monensin (BDGolgiStop) and Brefeldin A (GolgiPlug) were added for the last 6 h of cell incubation. As controls, cells were cultured in media only with Anti-human CD107 Ab. For the functional assay, cells were stained using the functional Ab panel (Additional file 1: Table S2) with similar staining protocol as for the phenotypic panel, but using the BD Cytofix/Cytoperm Fixation/Permeabilization Solution Kit (BD 554714) for intracellular staining.

### UMAP and phenoGraph analysis

BD FACSDiva software FCS3.0 files were imported into FlowJo software v10.7.2 followed by data cleaning to obtain good events using the FlowAI v2.3 plugin. An automated compensation matrix was generated based on the single-stained compensation beads and was applied to files. For manual gating and FACS data analysis, the flow cytometry gating strategies were done as shown in Additional file 2: Fig. S1 and Additional file 1: Tables S1 and S2 for each panel. For automated analysis, events were first down-sampled from the clean NK cell gate across all samples using FlowJo Downsample v3.3 plugin. Parameters such as age, sex, timepoint, group and SARS-CoV-2 Ab titers were annotated to each down-sampled population and then concatenated. The FlowJo UMAP v3.1 plugin was run on the resulting concatenated FCS file and the default settings were applied (distance function: Euclidean, nearest neighbors: 15, and minimum distance: 0.5) including all compensated parameters and SSC and FSC measurements. For cluster identification, the FlowJo PhenoGraph v3.0 plugin was run on the resulting UMAP with the default settings (nearest neighbors K = 30) and including the parameters Ki67, Perforin, Granzyme B, CD8, CD16, CD56, CD38, CD69, CCR7, CXCR3, CD127, CD161, CD25, CXCR5, NKG2C and CD57. For evaluation of marker expression changes in the distinct clusters, *z* score was calculated using the median fluorescence intensity (MFI) by subtracting the raw MFI score from the mean of sample distribution and dividing the results by the standard deviation.

### Measurements of anti-SARS-CoV-2 and anti-CMV antibodies

Serum samples were analyzed using the quantitative Elecsys® Anti-SARS-CoV-2 S (Roche Diagnostics) test on the Cobas 8000 e801pro platform for detection of antibodies to the SARS-CoV-2 spike protein receptor binding domain (RBD). The measuring range was between 0.4 and 250 U/mL. Threshold for positive results was set at ≥ 0.80 U/mL. Positive samples with Ab titers of > 250 U/mL were re-tested following a 1/10 dilution, and in applicable cases also following a 1/100 dilution which increased the upper level of measuring-range to 25,000 U/mL. Serological testing for the quantitative determination of antibodies to CMV in serum was done by chemiluminescent immunoassay using Liaison CMV IgG II assay and ran on the fully automated random access Liaison XL analyser. The assay was performed according to the recommendations of the manufacturer. Serum samples from healthy individuals were used in a dilution 1:3 with PBS supplemented with 0.5% BSA. IgG values were considered negative if < 12.0 U/mL, equivocal if ≥ 12–< 14.0 U/mL and positive if ≥ 14.0 U/mL.

### Statistical analysis

GraphPad Prism v9.1.0 (GraphPad Software) was used for statistical analysis. Differences between two unpaired groups were determined using a nonparametric Mann–Whitney U test. For evaluation of differences among three or more groups, a Kruskal–Wallis test followed by Dunn’s multiple comparison test were used in all the analysis. Correlations were evaluated in R v4.0.2 using Spearman’s rank correlation, with the use of the *rcorr* function from the package Hmisc v4.5. The adjusted p values were calculated with the corr.test function from *psych* v2.1.9 package using “fdr” option for multiple comparison adjustment. Correlation heatmap was graphically created with *corrplot* function from the package corrplot v0.1.3. All statistical details can be found in the figure legends including statistical tests used and exact value of n; i.e. number of samples analysed per timepoint.

## Results

### NK cell absolute counts, frequencies, subsets and phenotypes following BNT162b2 mRNA vaccination in healthy individuals and immunocompromised patients

In exploratory studies connected to the COVAXID BNT162b2 mRNA clinical trial (Bergman et al. 2021), we assessed total NK cells and their subsets in terms of absolute counts and frequencies, and cell phenotypes associated with effector function, activation, maturation, migration and differentiation before and after mRNA vaccination in healthy individuals and patients with primary and secondary immunodeficiencies (Fig. 1A; see Additional file 2: Fig. S1 for complete gating strategy). Details on the study cohort are provided in the Methods section and in Table 1.

In healthy individuals, as well as patients with primary and secondary immunodeficiencies, NK cell absolute counts remained constant overall throughout the clinical study period of 35 days (Fig. 1B, C). Likewise, no major perturbations in total NK cell frequencies were observed throughout the study period in healthy controls or in patients with primary or secondary immunodeficiencies (Fig. 1D). For comparison, results from frequency analyses of other major adaptive and innate immune cell populations are shown in Additional file 2: Fig. S2. Similar results were obtained when NK cell frequencies were assessed among major NK cell subgroups including CD56^bright^CD16^neg^ (referred to as CD56^bright^), CD56^bright^CD16^pos^, CD56^dim^CD16^pos^ (referred to as CD56^dim^) and CD56^dim^CD16^neg^ NK cells (Fig. 1E). Noteworthy, however, as expected due to differences in disease background (e.g., the primary immunodeficiency disorder [PID] group) or intervention combined with specific immunosuppressive treatment (e.g., the solid organ transplantation [SOT] group), NK cell absolute counts and frequencies differed among the group of healthy individuals and some of the patient groups (Fig. 1C–E).

**Fig. 2:**
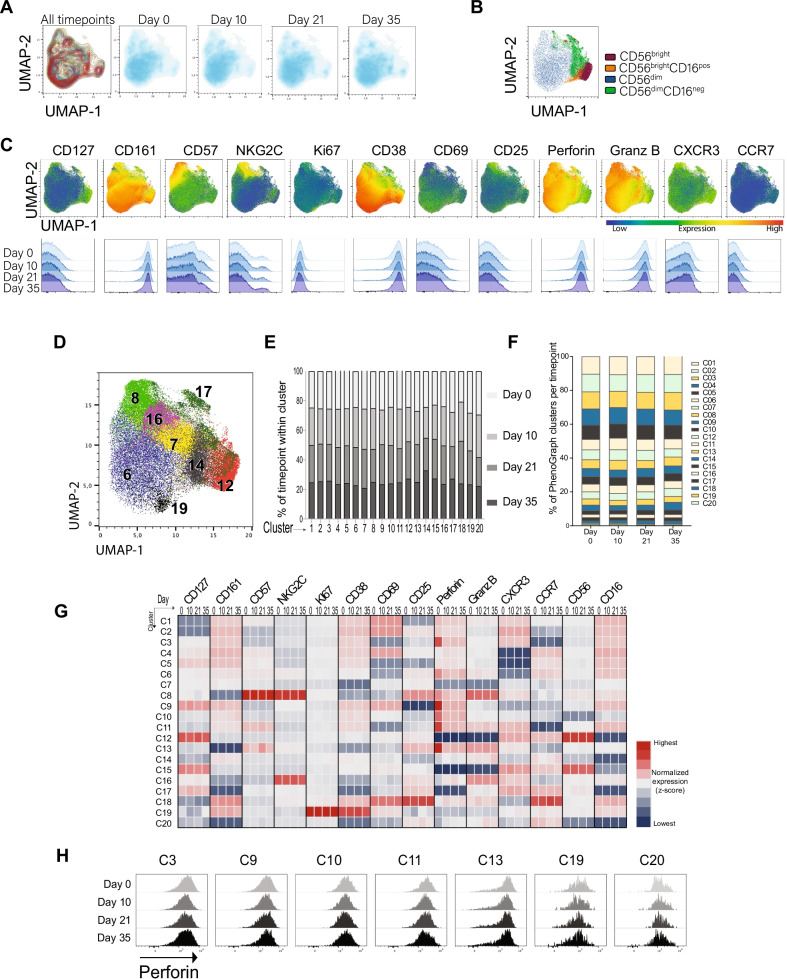
Manual and automated analysis of NK cells in healthy study subjects. **A** UMAP plots from manual gating analysis on concatenated files of total NK cells in healthy study subjects grouped together and separated by Days 0, 10, 21 and 35. **B** UMAP of CD56^bright^, CD56^bright^CD16^pos^, CD56^dim^ and CD56^dim^CD16^neg^ within pooled NK cells from healthy controls at all timepoints. **C** Expression of the indicated functional and phenotypic markers on total NK cells in the healthy control group represented in UMAP plots (upper panel) and corresponding histograms at Days 0, 10, 21 and 35 (lower panel). **D** Automated analysis showing UMAP of total NK cells depicting selected PhenoGraph overlaid clusters. **E** Distribution of the different timepoints analyzed (Days 0, 10, 21 and 35) within each PhenoGraph cluster. **F** Percentage of 20 PhenoGraph clusters within total NK cells at Days 0, 10, 21 and 35. **G** Heatmap of the mean fluorescence intensity (MFI) of the phenotypic markers used to characterize NK cells across PhenoGraph clusters at Days 0, 10, 21 and 35 (quantification of median expression values as column*-z* score). **H** Flow cytometry histograms showing expression of Perforin within the selected clusters. Healthy individuals (Day 0 n = 37, Day 10 n = 38, Day 21 n = 36, Day 35 n = 37)

Following these studies, NK cell characteristics throughout the sampling period were assessed with specific reference to the group of healthy individuals. A detailed phenotypic assessment by manual gating of total NK cells and their subsets including 26-parameter flow cytometry analyses (see Additional file 1: Table S2 for Ab panel and Additional file 2: Fig. S1A for gating strategy) revealed no major alterations over the four timepoints studied with respect to characteristics associated to maturation, differentiation, proliferation, activation, effector function, and migration (Fig. 2A–C). An additional attempt to verify the latter notion in healthy individuals was through an unsupervised approach using uniform manifold approximation and projection (UMAP) analysis. This revealed similar topological regions when comparing samples at different time points. When PhenoGraph analysis was applied to the samples, 20 distinct clusters were identified. All 20 clusters were equally distributed among the different timepoints (Fig. 2D–F). This visualization of the data also revealed expression of different markers and their distribution over time (Fig. 2G). A decrease in the expression of perforin from Day 0 to Day 10 was indicated in seven  clusters (clusters 3, 6, 8, 9, 10, 11, 13]. However, when this data was displayed using histograms in further evaluations of the observed patterns, decreased perforin expression was not as apparent (Fig. 2H). Altogether, the supervised and unsupervised data analysis supported the notion of largely preserved NK cell phenotypic characteristics throughout the sampling periods (Fig. 2A–H).

Taken together, these results revealed conserved NK cell numbers, frequencies, subsets, and phenotypes as assessed through consecutive peripheral blood samplings over 35 days following BNT162b2 mRNA vaccination.

### NK cell functional analyses following BNT162b2 mRNA vaccination in healthy individuals and immunocompromised patients

**Fig. 3:**
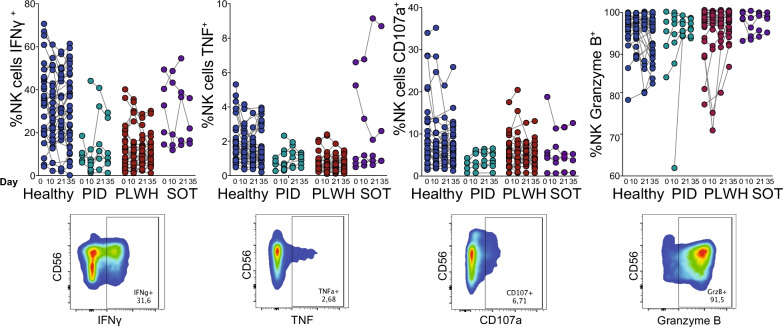
NK cell functional status at baseline and its correlation to anti-SARS-CoV-2 Ab titers. Functional characterization of total NK cells from healthy study subjects and select patient groups with immunodeficiency disorders receiving two doses of the BNT162b2 mRNA vaccine according to label. Samples at Days 0, 10, 21 and 35 show the frequencies of IFNγ-, TNF-, Granzyme B-, and CD107a-positive NK cells after 24 h of IL-12 (10 ng/ml) and IL-18 (100 ng/ml) stimulation (upper panel). Representative flow cytometry plots of functional markers expressed on NK cells (lower panel). Healthy controls (Day 0 n = 30, Day 10 n = 30, Day 21 n = 26, Day 35 n = 35), PLWH (Day 0 n = 25, Day 10 n = 24, Day 21 n = 24, Day 35 n = 23), PID (Day 0 n = 5, Day 10 n = 8, Day 21 n = 7, Day 35 n = 7), SOT (Day 0 n = 5, Day 10 n = 5, Day 21 n = 5, Day 35 n = 4). Statistical analysis was performed using a Kruskal–Wallis test followed by a Dunn’s multiple comparisons test. No statistical significance difference was observed within each respective group (i.e., when comparing days 0, 10, 21 and 35)

While the described data indicated a largely preserved NK cell compartment as assessed during the set peripheral blood sampling intervals, it remained possible that NK cells might be functionally altered after vaccination. Thus, total NK cells were stimulated for 24 h with interleukin-12 (IL-12) and interleukin-18 (IL-18) and analyzed for expression of intracellular interferon gamma (IFNγ), tumor necrosis factor (TNF), Granzyme B, as well as for cell surface expression of CD107 (See Additional file 1: Table S2 for Ab panel and Additional file 2: Fig. S1B for gating strategy). The studied NK cell functional parameters remained unaltered in the group of healthy individuals and in patients with primary or secondary immunodeficiencies over the course of the study period (Fig. 3). Similar to NK cell absolute counts and frequencies, NK cell functional parameters differed within the patient groups from what was observed in healthy controls (Fig. 3).

In line with the results on NK cell absolute counts and frequencies, these results revealed largely retained NK cell function as assessed through consecutive peripheral blood samplings over 35 days following BNT162b2 mRNA vaccination.

### Correlation analyses between baseline levels of NK cell variables and final SARS-CoV-2 antibody titers

**Fig. 4:**
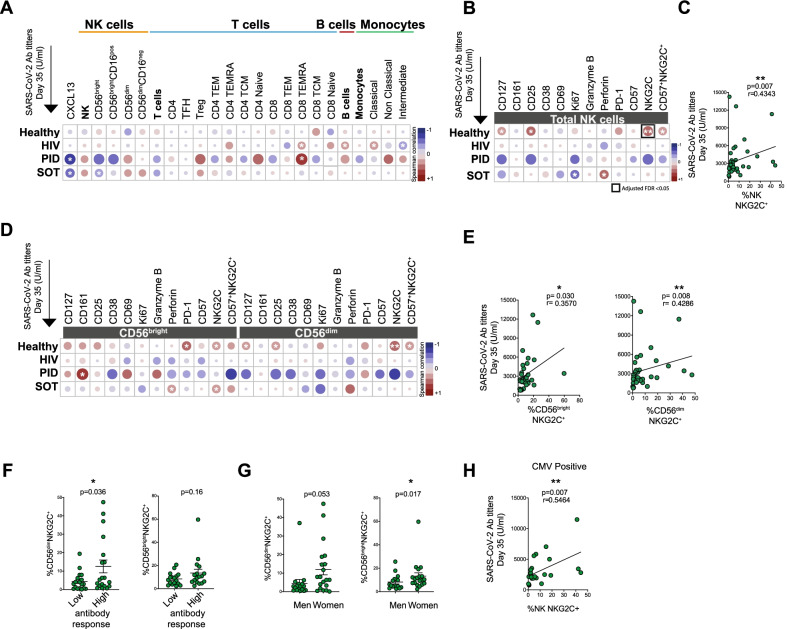
NK cell immune phenotypic characterization at baseline and its correlation to anti-SARS-CoV-2 Ab titers. **A** Heatmap displaying Spearman correlations between different lymphocytic (NK, T and B cells and their subsets), monocytic cell subsets, and markers expressed on total NK cells at baseline (Day 0) and anti-SARS-CoV-2 Ab titers at Day 35 in healthy study subjects and immunocompromised patients. Heatmaps depicting Spearman correlation between the expression of immune phenotypical markers at Day 0 on total NK cells (**B**), CD56^bright^ and CD56^dim^ cells **D** in healthy study subjects and immunocompromised patients, and the anti-SARS-CoV-2 Ab levels at Day 35. Spearman correlation between the NKG2C expression at Day 0 on total NK cells (**C**), CD56^bright^ and CD56^dim^ cells **E** in healthy study subjects and anti-SARS-CoV-2 Ab titers at Day 35 (n = 37). For the heatmaps diplaying Spearman correlation, false discovery rate (FDR) corrections were performed using the Benjamini–Hochberg test at an FDR < 0.05 significance threshold. Frequencies of NKG2C positive cells among CD56^dim^ and CD56^bright^ NK cells in healthy study subjects stratified into individuals with lower (50%) or higher (50%) anti-SARS-Cov-2 Ab titers **F** and in men and women **G** (n = 37). **H** Spearman correlation between the NKG2C expression at Day 0 on total NK cells and anti-SARS-CoV-2 Ab titers at Day 35 (n = 26) in healthy study subjects with a positive CMV serostatus. For Figs. 4A, B, D: Healthy individuals (n = 39), PLWH (n = 48), PID (n = 12), SOT (n = 34). For **C** and **E** Healthy individuals (n = 39). Mann–Whitney *U* test used to evaluate differences between two groups. Color in heatmaps indicates the *r* value, i.e. the strength of the correlation from the Spearman test, and asterisks show the statistical p value followed by FDR correction (highlighted in a black square if < 0.05). Significance level: *p < 0.05, **p < 0.01, ***p < 0.001

The results presented above allowed us to address whether NK cells, in any of a multitude of parameters assessed at baseline, affected the final outcome of SARS-CoV-2 Ab titers. To this end, neither frequencies of total NK cells, nor CD56^bright^, CD56^bright^CD16^pos^, CD56^dim^, nor CD56^dim^CD16^neg^ NK cells subsets correlated significantly with Ab titers at Day 35 as assessed in healthy individuals and in the groups of immunocompromised patients (Fig. 4A). For comparison, similar analyses are also shown for T cells and specific T cell subsets, B cells, as well as monocytes and specific monocytic subsets (Fig. 4A). NK cells were also stained for markers associated with NK cell maturation, differentiation, proliferation, activation and effector function. Among healthy individuals, a positive correlation between frequencies of NKG2C^+^ NK cells at baseline and Ab titers at Day 35 stood out (Figs. 4B, C). This correlation persisted when narrowed down to subsets of CD56^bright^NKG2C^+^ cells and CD56^dim^NKG2C^+^ cells (Figs. 4D, E). To address the correlation further, the healthy individuals were split into the 50% lowest and 50% highest Ab responders at Day 35. When doing this, NKG2C^+^ NK cell frequencies appeared to be more prominent in individuals with high anti-SARS-CoV-2 Ab response. Corroborating this finding, the participants with the most prominent CD56^dim^NKG2C^+^ expression were found in the high Ab group (Fig. 4F). This effect was particularly pronounced among women within the healthy study group (Fig. 4G), while no marked difference was observed between different age groups (data not shown). Enhanced numbers of CD56^dim^NKG2C^+^ cells are seen in individuals infected with human cytomegalovirus (HCMV) (Guma et al. 2004; Malmberg et al. 2012). When the healthy study subjects were assessed for HCMV serostatus, the association of NKG2C^+^ NK cells and SARS-CoV-2 Ab response at Day 35 was confirmed in the HCMV positive individuals (Fig. 4H).

These results indicate that an increased frequency of NKG2C^+^ “adaptive” NK cells at baseline may correlate with high Ab titers upon anti-SARS-CoV-2 BNT162b2 mRNA vaccination.

## Discussion

The present results reveal a preserved phenotype and functional capacity of NK cells in BNT162b2 mRNA-vaccinated healthy individuals and immunocompromised patients as assessed in the course of a prospective, open-label clinical trial (Bergman et al. 2021). The results indicate that NK cells are not significantly affected in terms of the studied gross parameters over time as a consequence of mRNA vaccination, at least not with the present BNT162b2 mRNA (Comirnaty®, Pfizer/BioNTech) vaccine assessed in a two-dose regimen with administration and dosing according to the label. Importantly, however, the results do not exclude an initial activation of NK cells over the first couple of days following vaccination. Indeed, during the first days after administration of the BNT162b2 mRNA vaccine, a noticeable increase in plasma IFNγ has been observed (Arunachalam et al. 2021), which may at least in part be a result of NK cell activation. Futhermore, outside of the present clinical trial, we had the possibility to follow in detail the NK cell responses in one anti-SARS-CoV-2 mRNA vaccinated index subject sampled regularly during the first 10 days after vaccination. In this index subject, markers associated with activation and functionality of NK cells were observed. These included CD69, IFNγ, TNF, granzyme B, and CD107 early following vaccination (< 5 days). Later responses (> 5 days) included increased expression of CXCR3 and proliferating NKG2C^+^ NK cells (unpublished observations). Similar early NK cells responses have also been seen in the course of vaccination with other types of vaccines (Marquardt et al. 2015). Although the results here presented do not exclude subtle vaccine-induced imprints, such as long-term signs of trained innate immunity (Netea et al. 2016), they do indicate that NK cells maintain their key phenotypic and functional properties throughout the course of vaccination. This is of importance not only in relation to the present BNT162b2 mRNA vaccine but also in relation to mRNA-based vaccines in general, as these vaccines currently are entering new indications in the prevention of other infectious diseases as well as in preventive and therapeutic contexts of cancer vaccination (Chakraborty et al. 2021). Therefore, it is of importance that immune effector cells such as NK cells will not be significantly perturbed by vaccination, particularly, in respect to their abilities to respond to future infection challenges and vaccination. The present results also argue against the idea that specific immune cells, or the “immune system” as such, are perturbated in a major way by vaccination.

Interestingly, the present study indicates a positive correlation between frequencies of NKG2C^+^ NK cells at baseline and anti-SARS-CoV-2 Ab titers two weeks after the second dose of vaccination. NKG2C^+^ NK cells were initially found to be expanded in humans infected with HCMV (Guma et al. 2004; Malmberg et al. 2012). NKG2C^+^ NK cell expansions have also been observed following diverse viral infections in humans, including hantavirus infection, chikungunya virus infection, HIV-1 infection, and notably severe COVID-19 (Bjorkstrom et al. 2021, 2011; Guma et al. 2006; Maucourant et al. 2020; Petitdemange et al. 2011). Notably, all these responses correlated with HCMV co-infection, suggesting that subclinical reactivation of HCMV could be one mechanism underlying the observed responses (Bjorkstrom et al. 2021). NKG2C^+^ NK cells are associated with specific enhanced anti-viral functional properties, including enhanced antibody-dependent cellular cytotoxicity (ADCC), and innate immune memory (Lee et al. 2015; Schlums et al. 2015). How CMV infection affects vaccination in context of NK cells has been a topic of discussion in recent years (Goodier et al. 2018).

In context of the present findings, it has been found that activated, but not resting, T cells can be recognized and killed by syngeneic NK cells (Rabinovich et al. 2003). Here, one may speculate that NKG2C^+^ NK cells act as a “set point” for T cell immune responeses. Such a model is in line with findings that NKG2C^+^ NK cells responded less well to activated autologous T cells than their NKG2C^−^ counter parts (Schlums et al. 2015). With this in mind, one could theoretically envision that reduced elimination of activated T cells enables enhanced Ab production through increased availability of T cell help for B cells. Studies in mice, in part, corroborate the findings above in that killing of CD8^+^ T cells by NK cells is dependent on NCR1/CD355 (Pallmer et al. 2019). This is interesting in relation to the fact that NKG2C^+^ adaptive NK cells often express low levels of NCRs such as NKp30 and NKp46 (Guma et al. 2004). The present observations on a positive correlation between NKG2C^+^ NK cell frequencies at baseline and anti-SARS-CoV-2-specific Ab titers clearly deserve further investigation in terms of mechanistic studies as well as independent confirmation in larger cohorts.

In relation to the present findings, it is also interesting to note that responsiveness to influenza vaccination was recently found to correlate with induced NKG2C-expression on NK cells following vaccination. It was found that a majority of responders displayed enhanced frequencies of NKG2C-expressing NK cells 7 or 14 days post-vaccination in comparison with low responders (Riese et al. 2020). Related to this, another study has demonstrated signs of long-term intracellular immune memory of human NK cells (Dou et al. 2015). It is also interesting to note that HBV vaccination has been shown to elicit an HBV-reactive subset of memory NK cells coexpressing CD57, CD69 and KLRG1 (Wijaya et al. 2021). Receptors involved in this specificity of NK cells for viral antigens are not fully defined.

One advantage of the present study is that it has been performed within the context of a well-controlled prospective, open-label clinical trial encompassing well-defined groups of study subjects, pre-defined study protocol, and external monitoring of the study. The present results do not exclude an imprint per se in the NK cell repertoire not captured with current methodology. For example, it cannot be excluded that NK cells might undergo distinct epigenetic programming that prepares an increased responsiveness following initial priming towards vaccine boost. Nor can induced long-term effects (months or years) on the NK cell repertoire following vaccination be excluded. Finally, it is well known that introduction of mRNA into many types of cells triggers innate immune responses (Zhang et al. 2019). It cannot be excluded that this could affect NK cells and thus leave an imprint in those cells affected. However, given that the present mRNA vaccine is administered locally into the deltoid muscle, it is unlikely that any significant amounts of vaccine-associated mRNA will reach circulating and tissue resident NK cell populations.

This study is limitated in context of abilities to study very early innate immune responses, given the pre-set sample periods (i.e., at Days 0, 10, 21 and 35) initially set to study adaptive immune responses. Furthermore, the studied patient groups are heterogeneous, both between and within the groups. The latter includes different diagnosis (e.g., within the PID group), interventions (e.g., different types of organ transplantations within the SOT group), and treatments (e.g., different types of immunosuppressive treatment in the SOT group), some of which affect the NK cell phenotype and function differently.

## Conclusions

The present results demonstrate largely preserved NK cell numbers, frequencies, subsets, phenotypes, and functions as assessed through extensive ex vivo analyses following consecutive peripheral blood samplings over 35 days in BNT162b2 mRNA vaccinated healthy individuals and immunosuppressed patients. Interestingly, a positive correlation between NKG2C^+^ NK cell subsets and anti-SARS-CoV-2 Ab titers was observed. The present results provide insights into NK cells in context of mRNA vaccinations against SARS-CoV-2, with relevance also for future mRNA vaccination against other viral infections and malignant diseases. Finally, they should also pave the way for deeper analyses of the effects of mRNA vaccination on NK cells.

## Supplementary Information

**Additional file 1: Table S1.** List of reagents used in the present study. **Table S2.** Antibodies used for Panel 1. **Table S3.** Antibodies used for Panel 2.

**Additional file 2: Figure S1. **Gating strategy for flow cytometry analysis, related to Figs. 1–4. **A** Gating strategy for phenotypic identification of lymphocytic and monocytic cell populations and subsets by flow cytometry in peripheral blood. **B** Gating strategy for identification of functional lymphocytic cell populations by flow cytometry in peripheral blood. **Figure S2.** Lymphocytic and monocytic cell frequencies after BNT162b2 mRNA vaccine administration, related to Fig. 1. **A** Frequency of lymphocytic (T and B cells and their subsets) and monocytic cell subsets at Days 0, 10, 21 and 35 in the healthy individuals and immunocompromised patients receiving two doses of the BNT162b2 mRNA vaccine according to label. Healthy individuals (Day 0 n = 37, Day 10 n = 38, Day 21 n = 36, Day 35 n = 37), PLWH (Day 0 n = 48, Day 10 n = 46, Day 21 n = 44, Day 35 n = 44), PID (Day 0 n = 12, Day 10 n = 16, Day 21 n = 14, Day 35 n = 12), SOT (Day 0 n = 34, Day 10 n = 33, Day 21 n = 33, Day 35 n = 30). Kruskal–Wallis test followed by a Dunn’s multiple comparisons test. No statistical significance difference was observed within each respective group (i.e., when comparing days 0, 10, 21 and 35).

## Data Availability

Requests for resources, reagents and further information can be made available upon request and should be directed to the lead contact, Hans-Gustaf Ljunggren (hans-gustaf.ljunggren@ki.se). This study did not generate new unique reagents and does not report original code.

## Abbreviations

Ab: Antibody
CD: Cluster of differentiation
CMV: Cytomegalovirus
COVID-19: Coronavirus disease 2019
CVID: Common variable immunodeficiency
DMSO: Dimethyl sulfoxide
EDTA: Ethylenediaminetetraacetic acid
FACS: Fluorescence-activated cell sorting
FCS: Forward scatter
HBV: Hepatitis B virus
HCMV: Human cytomegalovirus
HIV: Human immunodeficiency virus
IFNγ: Interferon gamma
IgA: Immunoglobulin A
IgG: Immunoglobulin G
IL-12: Interleukin 12
IL-18: Interleukin 18
MFI: Mean fluorescence intensity
MMF: Mycophenolate mofetil
mRNA: Messenger ribonucleic acid
NCR1: Natural cytotoxicity triggering receptor 1
NK: Natural killer
PBMC: Peripheral blood mononuclear cells
PBS: Phosphate buffered saline
PID: Primary immunodeficiencies
PLWH: People living with HIV
RBD: Receptor binding domain
RPMI: Roswell Park Memorial Institute
RRID: Research Resource Identifier
SARS-CoV-2: Severe acute respiratory syndrome coronavirus 2
SOT: Solid organ transplantation
TNF: Tumor necrosis factor
UMAP: Uniform manifold approximation and projection
