# Supplementary material for: NK cell frequencies, function and correlates to vaccine outcome in BNT162b2 mRNA anti-SARS-CoV-2 vaccinated healthy and immunocompromised individuals
Source: Mol Med. 2022 Feb 8;28:20. doi: 10.1186/s10020-022-00443-2 (PMC8822735; doi:10.1186/s10020-022-00443-2)
Supplement: Supplementary file 1 — Additional file 1: Table S1. List of reagents used in the present study. Table S2. Antibodies used for Panel 1. Table S3. Antibodies used for Panel 2. [file 10020_2022_443_MOESM1_ESM.docx]

**Additional file 1: TABLES**

**Table S1. List of reagents used in the present study**

| **REAGENT or RESOURCE** | **SOURCE** | **IDENTIFIER** |
| --- | --- | --- |
| **Antibodies** | | |
| Anti-human CD8 BUV395 (cloneRPA-T8) | BD Biosciences | Cat#563795; RRID: AB_2722501 |
| Anti-human CD16 BUV496 (Clone3G8) | BD Biosciences | Cat# 612928, RRID:AB_2870213 |
| Anti-human CD56 BUV563 (Clone NCAM16.2) | BD Biosciences | Cat# 612928, RRID:AB_2870213 |
| Anti-human CD38 BUV661 (CloneHIT2) | BD Biosciences | Cat# 612969, RRID:AB_2870242 |
| Anti-human CD69 BUV737 (CloneFN50) | BD Biosciences | Cat# 612817, RRID:AB_2870141 |
| Anti-human CD45 BUV805 (Clone HI30) | BD Biosciences | Cat# 612891, RRID:AB_2870179 |
| Anti-human CCR7 BV421 (Clone G043H7) | BioLegend | Cat# 353208, RRID:AB_11203894 |
| Anti-human CD15 V500 (CloneW6D3) | BioLegend | Cat# 323028, RRID:AB_2563400 |
| Anti-human CD45RA BV570(Clone HI100) | BioLegend | Cat# 304132, RRID:AB_2563813 |
| Anti-human CD57 BV605 (Clone QA17A04) | BioLegend | Cat# 393304, RRID:AB_2728426 |
| Anti-human CXCR3 BV650 (Clone G025H7) | BioLegend | Cat# 353730, RRID:AB_2563870 |
| Anti-human CD127 BV711 (Clone A019D5) | BioLegend | Cat# 351328, RRID:AB_2562908 |
| Anti-human CD4 BV750 (CloneSK3) | BD Biosciences | Cat# 566355, RRID:AB_2744426 |
| Anti-human CD3 BV785 (CloneSK7) | BD Biosciences | Cat# 563799, RRID:AB_2744384 |
| Anti-human CD14 BB700 (Clone MOP9) | BD Biosciences | Cat# 566465, RRID:AB_2739737 |
| Anti-human NKG2C PE-Vio615 (CloneREA205) | MILTENYI | Cat# 130-123-037, RRID:AB_2819445 |
| Anti-human CD161 PECy5 (Clone CX12) | BD Biosciences | Cat# 551138, RRID:AB_394068 |
| Anti-human CD25 PE-Cy5.5 (Clone BC96) | Thermo Fisher Scientific | Cat# 35-0259-42, RRID:AB_2744720 |
| Anti-human PD-1 PECy7 (CloneJ105) | Thermo Fisher Scientific | Cat# 25-2799-42, RRID:AB_10853804 |
| Anti-human CXCR5 APC-R700 (CloneRF8B2) | BD Biosciences | Cat# 565191, RRID:AB_2739103 |
| Anti-human CD19 APCFire 750 (Clone HIB19) | BioLegend | Cat# 302258, RRID:AB_2629691 |
| Anti-human Ki67 AF488 (Clone Ki-67) | BioLegend | Cat# 350508, RRID:AB_10933085 |
| Anti-human Perforin BB755 (Clone dG9) | BD Biosciences | Cat#6244361; NA |
| Anti-human Granzyme BB790 (Clone GB11) | BD Biosciences | Cat#624296; NA |
| Anti-human CD69 BUV395 (CloneFN50) | BD Biosciences | Cat# 564364, RRID:AB_2738770 |
| Anti-human CD56 BUV737 (Clone NCAM16.2) | BD Biosciences | Cat# 564447, RRID:AB_2744432 |
| Anti-human CD14 V500 (Clone M5E2) | BD Biosciences | Cat# 561391, RRID:AB_10611856) |
| Anti-human CD19 V500 (CloneHIB19) | BD Biosciences | Cat# 561125, RRID:AB_10563208 |
| Anti-human CD8 BV570 (Clone RPA-T8) | BioLegend | Cat# 301037, RRID:AB_10933259 |
| Anti-human CD3 BV650 (Clone OKT3) | BioLegend | Cat# 317324, RRID:AB_2563352 |
| Anti-human CD16 BV711 (Clone 3G8) | BioLegend | Cat# 302044, RRID:AB_2563802 |
| Anti-human CD4 APCH7 (Clone SK3) | BD Biosciences | Cat# 641398, RRID:AB_1645732 |
| Anti-IFNg BV421 (Clone 4SB34) | BioLegend | Cat# 502542, RRID:AB_2563882) |
| Anti-human TNFa PECy7 (Clone Mab11) | BD Biosciences | Cat# 557647, RRID:AB_396764 |
| Anti-human Granzyme B AF700 (Clone GB11) | BD Biosciences | Cat# 561016, RRID:AB_2033973 |
| **Chemicals, peptides, and recombinant proteins** | | |
| Lymphoprep | STEM CELL | 04-03-9391/02 |
| Complete RPMI 1640 medium | Gibco | Cat#31870-025, NA |
| 2-mM Glutamine | Invitrogen | Cat#25030149 |
| Penicillin and streptomycin | Invitrogen | Cat#15070063 |
| Brilliant stain buffer | BD Biosciences | Cat#563794 |
| LIVE/DEAD Fixable Aqua Dead cell stain kit | Thermo Fisher Scientific | Cat#L34957 |
| Bovine Serum Albumin | Sigma | Cat# A7030-100G, NA |
| eBioscience FoxP3/Transcription factor staining buffer set | Invitrogen | Cat#00-5523-00 |
| Fixation/permeabilization Solution Kit (Cytofix/Cytoperm) | BD Biosciences | Cat#554714 |
| BD GolgiStop | BD Biosciences | Cat#554724 |
| BD GolgiPlug | BD Biosciences | Cat#555029 |
| IL-12p70 | Peprotech | Cat#200-12-100 |
| IL-18 | MBL | Cat#B001.5 |
| **Critical commercial assays** | | |
| Liaison CMV IgG II | Diasorin | Cat#310745, NA |
| Elecsys Anti-SARS-CoV-2 | Roche Diagnostics | Cat# 09289267190 |
| **Software and algorithms** | | |
| FlowJo | FlowJo | v10.7.2 |
| GraphPad Prism | GraphPad | v9.1.0 |
| FACSDiva software | BD Biosciences | V8.0.1 |
| R | R Foundation | v4.0.2 |
| Hmisc (v4.5) | R environment | <https://cran.r-project.org/web/packages/Hmisc/index.html> |
| Corrplot (v0.1.3) | R environment | <https://cran.r-project.org/web/packages/corrplot/index.html> |
| Adobe Illustrator | Adobe | V25.4.1 |
| BioRender |  | <https://biorender.com/> |
| **Other** | | |
| Analyzer Cobas 8000 e801pro | Diagnostics Roche |  |
| BD FACSymphony | BD Biosciences |  |
| BD LSRFortessa | BD Biosciences |  |

**Table S2. Antibodies used for Panel 1**

| **LASER** | **FILTER** | **FLUOROPHORE** | **CLONE** | **ANTIBODY** | **DILUTION** |
| --- | --- | --- | --- | --- | --- |
| **EXTRACELLULAR STAINING** | | | | | |
| UV  355nm | 379/28 | BUV395 | RPA-T8 | **CD8** | 50 |
|  | 515/30 | BUV496 | 3G8 | **CD16** | 200 |
|  | 580/20 | BUV563 | NCAM16.2 | **CD56** | 200 |
|  | 670/25 | BUV661 | HIT2 | **CD38** | 25 |
|  | 735/30 | BUV737 | FN50 | **CD69** | 50 |
|  | 810/40 | BUV805 | HI30 | **CD45** | 25 |
| Violet  405nm | 450/50 | BV421 | QA17A04 | **CCR7** | 100 |
|  | 525/50 | V500 | W6D3 | **CD15** | 50 |
|  | 525/50 | V500 |  | **DCM** | 100 |
|  | 586/15 | BV570 | HI100 | **CD45 RA** | 200 |
|  | 605/40 | BV605 | QA17A04 | **CD57** | 50 |
|  | 677/20 | BV650 | G025H7 | **CXCR3** | 20 |
|  | 710/50 | BV711 | A019D5 | **CD127** | 50 |
|  | 750/30 | BV750 | SK3 | **CD4** | 50 |
|  | 810/40 | BV785 | SK7 | **CD3** | 100 |
| Blue 488nm | 710/50 | BB700 | MOP9 | **CD14** | 100 |
| Yellow/Green  561nm | 610/20 | PE-Vio615 | REA205 | **NKG2C** | 50 |
|  | 670/30 | PE-Cy5 | DX12 | **CD161** | 10 |
|  | 710/50 | PE-Cy5.5 | BC96 | **CD25** | 50 |
|  | 780/60 | PE-Cy7 | J105 | **PD1** | 50 |
| Red  639nm | 670/30 | APC | 3C10 | **Va7.2** | 50 |
|  | 730/45 | APC-R700 | RF8B2 | **CXCR5** | 50 |
|  | 780/60 | APCFire 750 | HIB19 | **CD19** | 200 |
| **INTRACELLULAR STAINING** | | | | | |
| Blue  488nm | 530/30 | AF488 | Ki-67 | **Ki67** | 100 |
|  | 750/30 | BB755 | dG9 | **Perforin** | 100 |
|  | 810/40 | BB790 | GB11 | **Granzyme B** | 100 |

**Table S3. Antibodies used for Panel 2**

| **LASER** | **FILTER** | **FLUOROPHORE** | **CLONE** | **ANTIBODY** | **DILUTION** |
| --- | --- | --- | --- | --- | --- |
| **EXTRACELLULAR STAINING** | | | | | |
| UV  355nm | 379/28 | BUV395 | FN50 | **CD69** | 200 |
|  | 735/30 | BUV737 | NCAM16.2 | **CD56** | 250 |
| Violet  405nm | 525/50 | V500 | M5E2 | **CD14** | 250 |
|  | 525/50 | V500 | HIB19 | **CD19** | 250 |
|  | 585/42 | Aqua |  | **DCM** | 100 |
|  | 610/20 | BV570 | RPA-T8 | **CD8** | 100 |
|  | 670/30 | BV650 | OKT3 | **CD3** | 20 |
|  | 710/50 | BV711 | 3G8 | **CD16** | 50 |
| Y/G 561nm | 661/20 | PE-Cy5 | DX12 | **CD161** | 10 |
| Red 636nm | 780/60 | APCH7 | SK3 | **CD4** | 10 |
| **INTRACELLULAR STAINING** | | | | | |
| Violet  405nm | 450/50 | BV421 | BC168 | **IL17** | 20 |
|  | 780/60 | BV785 | 4SB34 | **IFNg** | 100 |
| Y/G  561nm | 586/15 | PE | 3C10 | **Va7.2** | 50 |
|  | 780/60 | PE-Cy7 | Mab11 | **TNFa** | 20 |
| Red 639nm | 730/45 | AF700 | GB11 | **Granzyme B** | 100 |
