# Supplementary figures and images for: NK cell frequencies, function and correlates to vaccine outcome in BNT162b2 mRNA anti-SARS-CoV-2 vaccinated healthy and immunocompromised individuals
Source: Mol Med. 2022 Feb 8;28:20. doi: 10.1186/s10020-022-00443-2 (PMC8822735; doi:10.1186/s10020-022-00443-2)

**A**

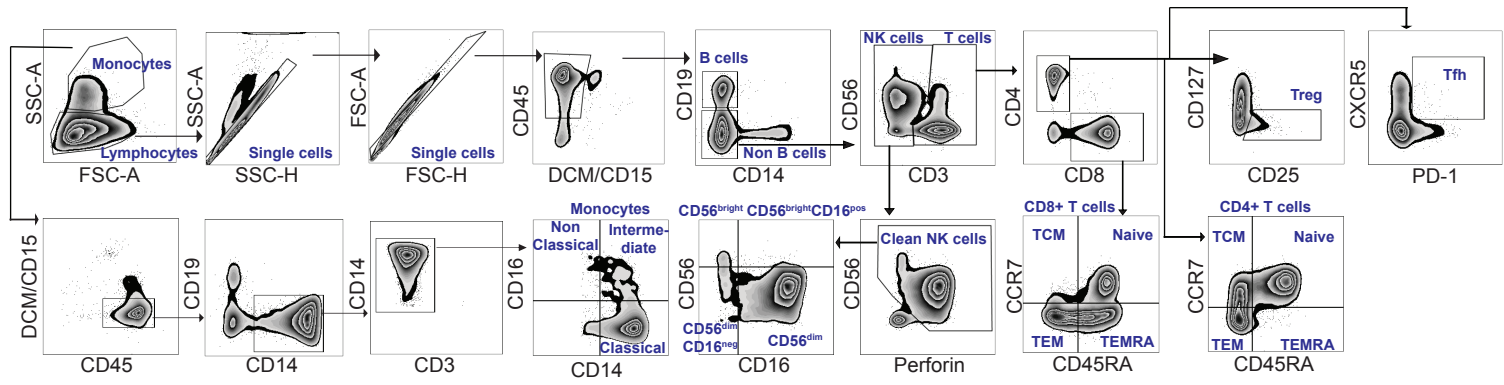

**B**

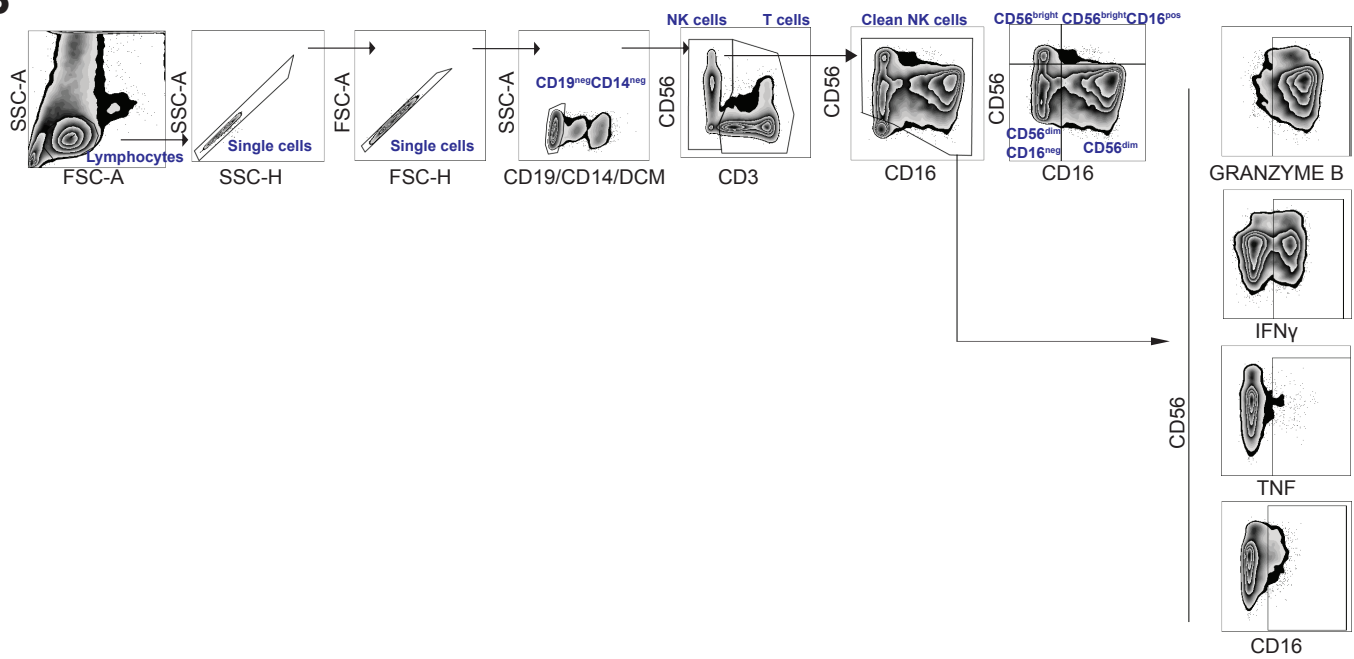

A

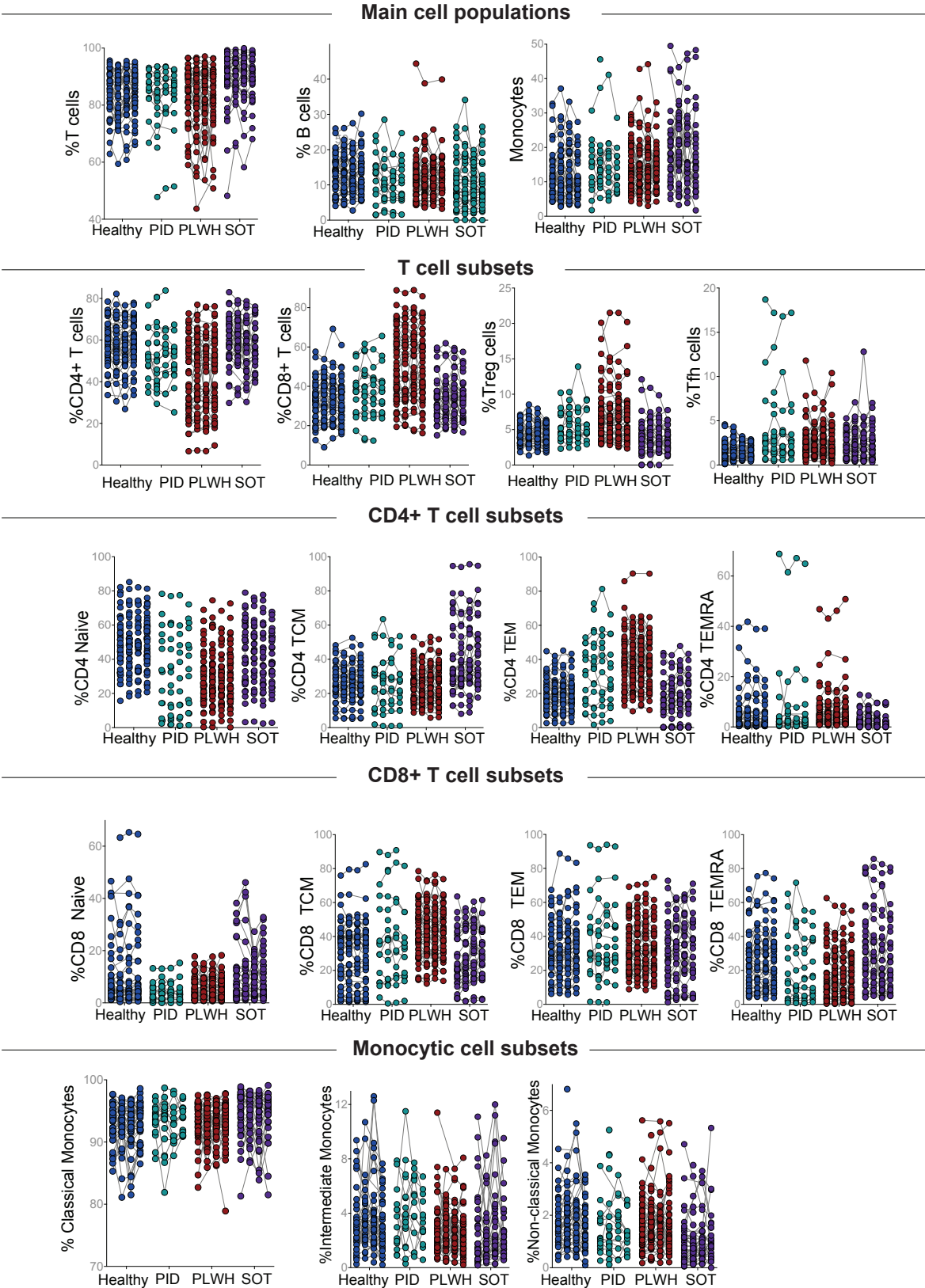

Supplement: Supplementary file 2 — Additional file 2: Figure S1. Gating strategy for flow cytometry analysis, related to Figs. 1–4. A Gating strategy for phenotypic identification of lymphocytic and monocytic cell populations and subsets by flow cytometry in peripheral blood. B Gating strategy for identification of functional lymphocytic cell populations by flow cytometry in peripheral blood. Figure S2. Lymphocytic and monocytic cell frequencies after BNT162b2 mRNA vaccine administration, related to Fig. 1. A Frequency of lymphocytic (T and B cells and their subsets) and monocytic cell subsets at Days 0, 10, 21 and 35 in the healthy individuals and immunocompromised patients receiving two doses of the BNT162b2 mRNA vaccine according to label. Healthy individuals (Day 0 n = 37, Day 10 n = 38, Day 21 n = 36, Day 35 n = 37), PLWH (Day 0 n = 48, Day 10 n = 46, Day 21 n = 44, Day 35 n = 44), PID (Day 0 n = 12, Day 10 n = 16, Day 21 n = 14, Day 35 n = 12), SOT (Day 0 n = 34, Day 10 n = 33, Day 21 n = 33, Day 35 n = 30). Kruskal–Wallis test followed by a Dunn’s multiple comparisons test. No statistical significance difference was observed within each respective group (i.e., when comparing days 0, 10, 21 and 35). [file 10020_2022_443_MOESM2_ESM.pdf]
